# Upper thoracic spine mobilization and mobility exercise versus upper cervical spine mobilization and stabilization exercise in individuals with forward head posture: a randomized clinical trial

**DOI:** 10.1186/s12891-017-1889-2

**Published:** 2017-12-12

**Authors:** Juchul Cho, Eunsang Lee, Seungwon Lee

**Affiliations:** 1 Department of Physical Therapy, Graduate School of Sahmyook University, 815, Hwarang-ro, Nowon-gu, Seoul, South Korea; 2 Department of Physical Therapy, Sahmyook University, 815, Hwarang-ro, Nowon-gu, Seoul, South Korea

**Keywords:** Mobilization, Therapeutic exercises, Forward head posture, Neck pain

## Abstract

**Background:**

Although upper cervical and upper thoracic spine mobilization plus therapeutic exercises are common interventions for the management of forward head posture (FHP), no study has directly compared the effectiveness of cervical spine mobilization and stabilization exercise with that of thoracic spine mobilization and mobility exercise in individuals with FHP.

**Methods:**

Thirty-two participants with FHP were randomized into the cervical group or the thoracic group. The treatment period was 4 weeks, with follow-up assessment at 4 and 6 weeks after the initial examination. Outcome measures including the craniovertebral angle (CVA), cervical range of motion, numeric pain rating scale (NPRS), pressure pain threshold, neck disability index (NDI), and global rating of change (GRC) were collected. Data were examined with a two-way repeated-measures analysis of variance (group × time).

**Results:**

Participants in the thoracic group demonstrated significant improvements (*p* < .05) in CVA, cervical extension, NPRS, and NDI at the 6-week follow-up compared with those in the cervical group. In addition, 11 of 15 (68.8%) participants in the thoracic group compared with 8 of 16 participants (50%) in the cervical group showed a GRC score of +4 or higher at the 4-week follow-up.

**Conclusions:**

The combination of upper thoracic spine mobilization and mobility exercise demonstrated better overall short-term outcomes in CVA (standing position), cervical extension, NPRS, NDI, and GRC compared with upper cervical spine mobilization and stabilization exercise in individuals with FHP.

**Trial registration:**

KCT0002307, April 11, 2017 (retrospectively registered).

## Background

Many people living in modern society experience neck pain. Moreover, the increasing use of smartphones has resulted in increasing incidence of neck pain [1–3]. During smartphone use, the user’s neck is more bent than when looking at video display terminals in general [4], and neck pain can occur because the cervical extensor becomes activated and the load on the erector spinae increases in order to adjust the neck balance [4]. Additionally, touching the screen and using the smartphone for a long time without supporting the arm causes fatigue of the neck and shoulder, and increases the load on the cervical spine [5]. Continuous load on the cervical spine leads to variations in the spinal curve, resulting in degenerative change of joints, a straight cervical spine, and forward head posture (FHP) [6–8], which can worsen and progress to cervical herniation of the intervertebral disc [9]. FHP is defined as increased extension of the upper cervical spine and increased flexion of the lower cervical spine and upper thoracic spine, with the head position around the sagittal plane showing forward deviation from the gravity line [10, 11]. In the FHP position, the loads applied to the muscles around the neck and shoulder are 3.6 times the loads in the normal position [12]. Particularly, FHP causes shortening of the sternocleidomastoid, scalenus anterior, and upper trapezius muscles, and lengthening of the levator scapulae and semispinalis capitis muscles, leading to abnormal activation of the cervical spine flexor and extensor muscles [13].

Previous studies reported that 60% of patients with neck and shoulder pain presented with FHP [14], and FHP caused asymmetric muscle activation in the spine (which is an important indicator of neck pain), restricted functional activity, and caused spine deviation and lateral inclination of the pelvis [15–17]. Cervical instability results from hypomobility of the upper cervical spine and upper thoracic spine and hypermobility of the lower cervical spine. Particularly, the protective action of movement and the reaction force is decreased by the induced change in vertebral structures [18]. Therefore, most clinicians examine the thoracic spine in individuals with neck pain [19, 20]. Dysfunction of the lower cervical spine and articular disc lesions can be causes of pain in the upper thoracic spine, and dysfunction of the upper thoracic spine restricts the movement of the cervical spine and causes pain [21, 22]. These biomechanical relations of the cervical spine and the thoracic spine are related to movement and become important factors causing neck pain [23]. Some studies report that neck pain and range of motion were improved by applying manual therapy on the upper thoracic spine and on the cervical spine of patients with neck pain, thus improving their movement [24, 25].

In clinical practice, physical therapists generally use different modalities, therapeutic exercises, non-thrust mobilization, and thrust manipulation as representative interventions to improve neck pain and FHP [26–29]. A review of the literature highlights recent studies that investigated interventions with manual therapy and active exercise in acute and chronic cervical diseases [30, 31]. Particularly, it was proposed that a combination of manual therapy and therapeutic exercise is effective for the management of mechanical neck pain [28]. There are two types of manual therapy: joint mobilization and joint manipulation [20, 32]. However, previous studies suggested that adverse effects, such as local discomfort, headache, dizziness, and malaise were fewer in mobilization than in manipulation [33, 34]. A study on joint mobilization and manipulation therapy reported that the two types of therapies showed equivalent efficacy [35].

Although many studies have been conducted on patients with neck pain, there is insufficient evidence on the effectiveness of the combination of joint mobilization and therapeutic exercise in individuals with FHP. Therefore, the purpose of this study was to identify the effect of the combination of joint mobilization and therapeutic exercises in improving pain and movement in individuals with FHP. In addition, although most previous studies focused on the cervical spine, this study aimed to identify the effect of intervention on the upper thoracic spine.

## Methods

### Participants

In this study, 32 participants with neck pain from FHP were recruited in the Department of Rehabilitation Medicine of K University Hospital from November to December 2016. The following inclusion criteria were applied: participants should have a primary complaint of neck pain (pain between the posterior part of the cervical spine and the interscapular region), age from 20 to 29 years (age at which there is no degenerative change and deformation of the cervical vertebrae), and symptoms of FHP. For the diagnosis of FHP, the craniovertebral angle (CVA) was evaluated. Participants were excluded if they presented any serious pathology such as tumor, had history of whiplash injury within 3 months of the examination, underwent prior surgery to the cervical or thoracic spine, and exhibited positive neurologic signs consistent with nerve root compression. The protocol for the study was approved by the Institutional Review Board of Sahmyook University. All participants provided informed consent before their participation in the study.

### Examination procedures

All participants underwent demographic information collection and physical examination. The cervical spine and thoracic spine were evaluated in the sitting and prone positions, and a passive physiological intervertebral motion test (which used physiological movements such as flexion, extension, lateral-flexion, and rotation) and a passive accessory intervertebral motion test were conducted [20, 36]. Spurling’s test, compression test, distraction test, and upper limb tension tests were performed as special tests for neurological signs and symptoms [32]. The study period lasted for 6 weeks. Evaluations were conducted before and after treatment. Each intervention was applied 10 times for 4 weeks, and follow-up evaluation was conducted after 2 weeks. The cervical group performed upper cervical mobilization and cervical retraction exercise. The thoracic group performed upper thoracic mobilization and upper thoracic extension exercise. The interventions were administered by one physical therapist who had a clinical experience of 13 years and has completed >300 h of the manual therapy education course.

### Randomization

**Fig. 1:**
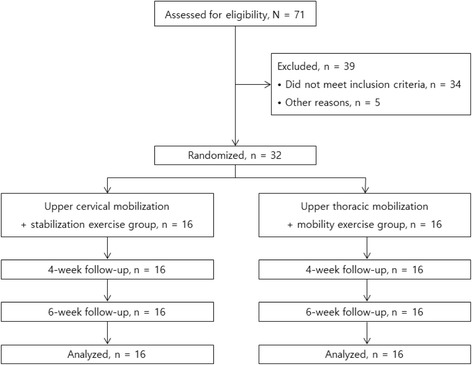
Flowchart of the recruitment, randomization, and follow-up of participants

One of three physical therapists recruited participants who met the eligibility criteria, and collected all data on baseline characteristics of participants. After the baseline examination, the 32 participants were randomly assigned to either the cervical group (*n* = 16) or the thoracic group (n = 16) by selecting one of two cards from a box (Fig. 1). The participants were not informed of the research hypothesis, and the evaluator was blinded to the participants’ assignment to the treatment groups. All participants were instructed not to discuss any information related to the studied treatments with the other participants.

### Cervical group

The participant sat in the correct posture on a chair with back rest, and the therapist stood facing the left trunk of the participant to apply the mobilization improving the flexion of the upper cervical spine (C1–2) [32]. The therapist covered the rear of the cervical part of the participant with his right hand for stabilization, and placed his thumb and index fingers on the atlas of the participant. To conduct the atlanto-occipital mobilization, the therapist placed his left hand at the right side of the participant and at the same time placed his fifth finger under the occipital area and pulled the participant’s head toward his trunk. C1–2 segmental mobilization was conducted to improve flexion according to the convex rule by moving the occipital condyle of the participant to the backward direction by using the trunk and left hand of the therapist. That is, the therapist made the patient increase the inward motion of the chin. After joint mobilization was conducted on one occipital condyle, the same procedure was applied to the other side. The discomfort of the participant was checked during the joint mobilization, which was conducted for 30 s, three times for each segment. The mobilization lasted for a total of <5 min, with a 10 s break between the mobilizations of each joint. The total time of intersegmental movement and break intervals was 30 s.

**Fig. 2:**
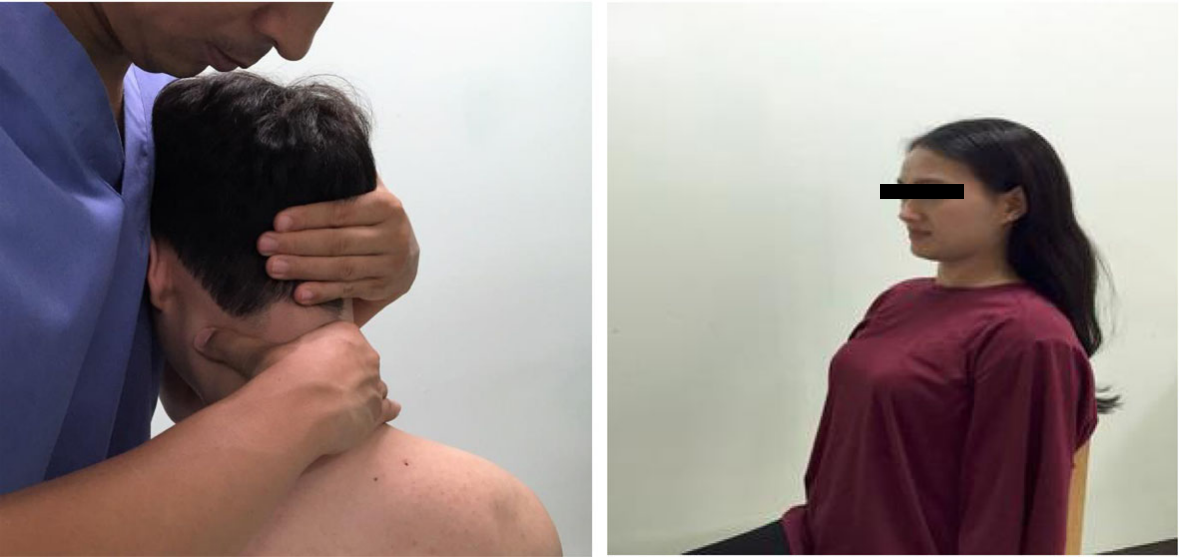
Upper cervical (C1–2) spine mobilization and stabilization exercise used in this study

The cervical retraction exercise that was used in a previous study was modified and performed as the cervical spine stabilization exercise in this study [37]. The participants performed cervical retraction by breathing in until gaining the feeling that the occipital bone was pushed back, while sitting straight on a chair with back rest. During the performance of the exercise, the therapist re-educated the participant through the palpation and observation of cervical segments in order not to generate compensation. A total of three sets were conducted, with one set consisting of repeating the motion that was maintained for 10 s up to 10 times. A break of 5 s was provided per one movement and 30 s per one set. Therefore, the total exercise time was <10 min (Fig. 2).

### Thoracic group

The participants were placed in the prone position and the therapist stood facing the joint where the mobilization will be applied to improve the extension of the upper thoracic spine (T1–2) [32]. The index and middle fingers of the left hand of the therapist were placed on the vertebral transverse process of the participant, which was located in the caudal side of the targeted segment. After the observation of the movement by palpating the area between the spinous processes of selected segments by using the index finger of the right hand of the therapist, the lateral side of the right palm of the therapist was placed on the index and middle fingers of his left hand, and thoracic segmental mobilization was conducted from ventral to caudal direction. Additional wedge was also used to generate exact and strong local movement in the targeted segment. The intervention time was the same as in the cervical group.

**Fig. 3:**
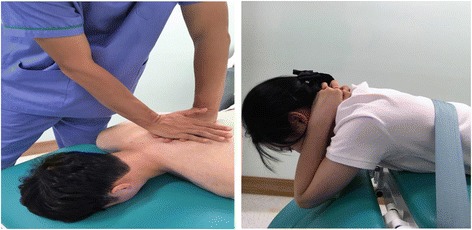
Upper thoracic (T1–2) spine mobilization and mobility exercise used in this study

Upper thoracic extension exercise was conducted by correcting and supplementing the prone trunk lift that was used in a previous study in order to improve the thoracic mobility [38]. The participant covered his or her cervical spine with both hands and lifted his or her upper body slowly, until gaining the feeling that the occipital bone was pushed upward after moving the chin inward. During the performance of the movement, the therapist re-educated the participant through the palpation and observation of upper thoracic segments. The intervention time was the same as in the cervical group (Fig. 3).

### Outcome measures

Primary outcome measure consisted of the CVA, which was measured using the photograph of the profile of each participant. The participants were made to maintain the natural head posture through the measurement method of self-balance posture [39]. The CVA was the angle made by the line that connects the seventh cervical spine with the tragus and the horizontal line of the seventh cervical spine in the sitting and standing positions, and a CVA of <49° was categorized as FHP in previous studies [40, 41]. In a previous study, CVA measurement was reported to have a high reliability [42].

The secondary outcome measures included the cervical range of motion (CROM), numeric pain rating scale (NPRS), pressure pain threshold (PPT), neck disability index (NDI), and global rating of change (GRC). A CROM device (Deluxe inclinometer; Performance Attainment Associates and Mednet Technologies Inc., Roseville, MN, USA) was used to test the active range of motion of the cervical spine. The highest intra-tester reliability (intra-class correlation coefficient [ICC] = 0.87–0.96) for patients with neck pain was identified [43], and the minimal detectable change (MDC) of the CROM had a variation of 5° to 10° [44, 45]. To measure intensity of their current pain, we used an 11-point NPRS, and the NPRS has been shown to have high reliability and validity in previous studies. [46, 47]. An MDC of 2.1 points, with a minimal clinically important difference (MCID) of 1.3 points, was indicated for patients with neck pain [48]. A pressure algometer (J-Tech 12–1485 Commander Algometer; JTECH Medical, Midvale, UT, USA) was used to identify the change of the PPT after the therapeutic intervention. PPT was measured by targeting the upper fibers of the trapezius when the participants were in the sitting position. In pressure algometry, the intra-rater reliability (ICC = 0.94–0.97) and inter-rater reliability (ICC = 0.79–0.90) were identified for patients with neck pain, and it was indicated that the MDC was 47.2 kPa [49]. The NDI was used to evaluate the disability index of the cervical spine [50]. The NDI has been demonstrated to be a valid evaluative tool for patients with neck pain. In a recent systematic review of the literature, it was concluded that the NDI has an MDC of 10%, with an MCID of 14% [51]. The GRC was used to assess the patient satisfaction after the intervention only. [52]. GRC is used as an objective means to test the improvement of patient symptoms, to identify the intervention effect after treatment completion in clinical practice, and this scale has been shown to have reliability and validity in previous studies. The MCID has been reported as a 3-point change from the baseline value [53]. All outcome measures were collected by an assessor blinded to the group assignments.

### Sample size

The number of required participants was calculated using G*Power 3.1 [54]. This study was set according to effect size f(V) 0.84 of the NPRS in a previous study [55] that compared the effect of manipulation of the cervical and the thoracic spine of patients with neck pain. The sample size was statistically calculated using analysis of variance (ANOVA), with the following features: repeated measures, within–between interaction, power (1-β err prob) 0.80, and alpha level .05. Allowing for a conservative dropout rate of 20%, we planned to recruit at least 32 participants in this study.

### Data analysis

The baseline characteristics of participants were compared between treatment groups, using independent *t* tests and chi-square tests to assess the adequacy of the randomization (Table 1). The effects of interventions on posture, mobility, and pain was examined by use of a two-way repeated-measures ANOVA, with the treatment group (cervical spine vs. thoracic spine) as the between-participant variable and time (baseline, 4 weeks follow-up, and 6 weeks follow-up) as the within-participant variable. In addition, GRC score for the between groups were compared using an independent *t* test. Data analysis was conducted using SPSS (version 18.0; SPSS Inc., Chicago, IL, USA) statistical software. Statistical significance was set at *p* < .05.

**Table 1:** Baseline demographics for the two groups

Baseline variable	Cervical group (*n* = 16)^a^	Thoracic group (*n* = 16)^a^	*p*-Value
Age (years)	23.8 (2.6)	23.9 (3.1)	.951^†^
Sex (female), number (%)	12 (75.0)	11 (68.8)	.694^‡^
Height (cm)	164.0 (6.3)	165.8 (7.9)	.495^†^
Weight (kg)	57.0 (11.3)	56.3 (10.6)	.861^†^
Duration of symptoms (months)	32.9 (26.0)	24.8 (30.0)	.420^†^

^a^Values are mean (standard deviation)

^†^Independent t test

^‡^Chi-square

## Results

The baseline characteristics were similar between the groups for all variables (*p* > .42) (Table 1). The within-group change scores and between-group differences along with 95% confidence intervals (CI) for all outcome measures can be found in Tables 2 and 3. The participants reported no adverse events during the treatment period, nor were any identified during the 6-week follow-up.

**Table 2:** Within-group change score and pairwise comparisons of between-group change scores for cervical range of motion

Measure/group	Baseline^a^	4 Weeks^a^	6 Weeks^a^	Mean within-group differences		Mean between-group differences	
				Baseline to 4 weeks^b^	Baseline to 6 weeks^b^	Baseline to 4 weeks^b^	Baseline to 6 weeks^b^
Flexion (°)							
Cervical group	49.3 (10.1)	57.1 (9.3)	53.2 (11.5)	7.8 (2.8, 12.8)	3.9 (−0.8, 8.7)	2.2 (−3.4, 7.7); *p* = .428	4.7 (−1.0, 10.4); *p* = .104
Thoracic group	50.6 (9.5)	60.6 (9.0)	59.3 (7.4)	10.0 (7.1, 13.0)	8.6 (5.0, 12.2)		
Extension (°)							
Cervical group	63.3 (8.4)	69.6 (8.1)	64.6 (7.3)	6.3 (2.4, 10.2)	1.3 (−4.0, 6.6)	3.9 (−0.9, 8.8); *p* = .108	6.2 (1.2, 11.2); *p* = .016
Thoracic group	59.7 (9.5)	70.0 (9.0)	67.2 (8.0)	10.3 (5.2, 15.3)	7.5 (3.7, 11.3)		
Right lateral flexion (°)							
Cervical group	39.8 (5.7)	45.3 (5.3)	42.9 (4.9)	5.6 (3.0, 8.2)	3.2 (0.0, 6.3)	1.2 (−2.5, 4.9); *p* = .515	−0.4 (−4.6, 3.8); *p* = .833
Thoracic group	37.6 (6.1)	44.4 (2.9)	40.4 (4.4)	6.8 (4.0, 9.6)	2.8 (−0.3, 5.8)		
Left lateral flexion (°)							
Cervical group	41.9 (6.0)	45.8 (5.6)	42.4 (4.9)	3.9 (2.0, 5.9)	0.5 (−2.2, 3.2)	3.4 (0.3, 6.5); *p* = .033	3.5 (−0.1, 7.0); *p* = .051
Thoracic group	37.2 (7.5)	44.5 (4.9)	41.2 (5.0)	7.3 (4.7, 9.9)	4.0 (1.6, 6.5)		
Right rotation (°)							
Cervical group	67.4 (8.9)	76.3 (5.7)	74.3 (7.0)	8.9 (5.3, 12.6)	6.9 (3.7, 10.0)	4.6 (−0.7, 1.0); *p* = .088	0.8 (−5.2, 6.7); *p* = .797
Thoracic group	63.7 (12.0)	77.3 (6.5)	71.3 (8.6)	13.6 (9.4, 17.8)	7.6 (2.3, 12.9)		
Left rotation (°)							
Cervical group	69.1 (8.1)	78.3 (5.3)	75.2 (6.3)	9.1 (5.0, 13.3)	6.1 (1.9, 10.2)	5.9 (−0.1, 12.0); *p* = .054	3.3 (−3.9, 10.4); *p* = .054
Thoracic group	63.6 (13.4)	78.6 (8.3)	72.9 (10.3)	15.1 (10.3, 19.8)	9.3 (3.1, 15.4)		

^a^Values are mean (standard deviation)

^b^Values are mean (95% confidence interval)

**Table 3:** Outcome data for craniovertebral angle, neck pain, pain sensitivity, and disability

Measure/group	Baseline^a^	4 Weeks^a^	6 Weeks^a^	Mean within-group differences		Mean between-group differences	
				Baseline to 4 weeks^b^	Baseline to 6 weeks^b^	Baseline to 4 weeks^b^	Baseline to 6 weeks^b^
CVA (sitting)							
Cervical group	45.1 (3.9)	50.6 (4.4)	48.4 (5.7)	5.4 (2.9, 7.9)	3.3 (0.1, 6.4)	0.9 (−2.1, 4.0); *p* = .536	2.1 (−1.6, 6.0); *p* = .252
Thoracic group	43.6 (3.8)	50.0 (3.8)	49.1 (3.0)	6.4 (4.4, 8.4)	5.4 (3.0, 8.0)		
CVA (standing)							
Cervical group	50.6 (4.8)	52.0 (5.7)	51.3 (5.2)	1.4 (−1.2, 3.9)	0.6 (−1.9, 3.1)	4.3 (1.2, 7.4); *p* = .008	3.3 (0.1, 6.4); *p* = .042
Thoracic group	48.4 (4.6)	54.1 (4.3)	52.3 (3.3)	5.7 (3.7, 7.7)	3.9 (1.8, 6.0)		
NPRS (0–10)							
Cervical group	3.6 (1.4)	2.3 (1.0)	2.3 (1.0)	1.3 (0.8, 1.8)	1.3 (0.7, 1.9)	1.3 (0.6, 2.1); *p* < .001	1.4 (0.6, 2.3); *p* = .002
Thoracic group	4.2 (1.5)	1.6 (0.8)	1.4 (0.7)	2.6 (2.0, 3.2)	2.8 (2.0, 3.4)		
PPT (kPa)							
Cervical group	35.9 (8.2)	48.4 (10.5)	46.8 (10.0)	12.5 (9.7, 15.3)	10.8 (8.2, 13.4)	1.9 (−1.4, 5.2); *p* = .251	−2.6 (−7.0, 1.9); *p* = .251
Thoracic group	36.3 (12.7)	50.6 (12.1)	44.5 (11.2)	14.4 (12.5, 16.3)	8.3 (4.4, 12.1)		
NDI (0–50)							
Cervical group	7.9 (4.3)	5.4 (3.7)	5.3 (3.9)	2.4 (1.3, 3.6)	2.6 (1.6, 3.7)	3.3 (1.0, 5.6); *p* = .006	3.4 (1.0, 5.8); *p* = .008
Thoracic group	10.4 (5.0)	4.6 (2.2)	4.3 (2.0)	5.8 (3.7, 7.8)	6.1 (3.8, 8.3)		

*CVA* craniovertebral angle, *NPRS* numerical pain rating scale, *PPT* pressure pain threshold, *NDI* neck disability index

^a^Values are mean (standard deviation)

^b^Values are mean (95% confidence interval)

In this study, the active cervical extension showed a significant group-by-time interaction (F_2,29_ = 3.882, *p* = .026, *η*_p_^2^ = .115), with the thoracic group indicating significantly (t_29_ = 2.54, *p* = .016) better improvement in active cervical extension (7.5°; 95% CI: 3.7, 11.3) over time than those in the cervical group (1.3°; 95% CI: -4.0, 6.6). However, no significant group-by-time interaction was observed for CROM, as measured using flexion, lateral flexion, and rotation. The CVA (standing position) showed a significant group-by-time interaction (F_2,29_ = 4.549, *p* = .014, *η*_p_^2^ = .132), with the thoracic group indicating significantly (t_29_ = 2.13, *p* = .042) better improvement in CVA (3.9°; 95% CI: 1.8, 6.0) over time than those in the cervical group (0.6°; 95% CI: -1.9, 3.1). However, the CVA (sitting position) demonstrated no significant group-by-time interaction. The NPRS showed a significant group-by-time interaction (F_2,29_ = 9.779, *p* = .001, *η*_p_^2^ = .246), with the thoracic group indicating better pain reduction over time than those in the cervical group. However, no significant group-by-time interaction was observed for PPT, as measured using a pressure algometer. The NDI showed a significant group-by-time interaction (F_2,29_ = 7.938, *p* = .004, *η*_p_^2^ = .209), with the thoracic group indicating better improvement in disability index over time than those in the cervical group. In case of GRC after 4 weeks, the thoracic group demonstrated significantly (t_29_ = 2.725, *p* = .011) better improvements on the GRC measure (mean ± standard deviation, +4.25 ± 1.06) than the cervical group (+3.37 ± 0.72), with a mean difference between groups of 0.87 points (95% CI: 0.2, 1.5).

## Discussion

The results of this study corresponded with those of a previous research that shows the efficacy of manipulation and mobilization of the cervical and thoracic spine in patients with neck pain [24, 56, 57]. The novelty of the current study is that the results suggest that the combination of upper thoracic mobilization and mobility exercise may provide short-term benefits to individuals with FHP.

FHP results in deformation of joints due to poor postures for a long time. Mobilization as treatment was conducted to improve the flexion of the upper cervical spine and to enhance the extension of the upper thoracic spine [32]. According to the purpose of the mobilization, this study showed improvement of the CROM in both groups; however, there was a significant difference between the two groups in cervical spine extension. Previous studies also showed the increase of the range of motion by improving joint hypo-mobility and the adhesion between soft tissues when the joint mobilization technique was applied to patients with mechanical neck pain [23, 58]. Particularly, it was reported that there were more improvements of movement limitation in patients with the most serious pain. In the case of therapeutic exercise, in this study, the stabilization exercise was conducted in the lower cervical spine and the mobility exercise was performed in the upper thoracic spine. The stabilization exercise for the cervical spine was a low-intensity isometric exercise, and the mobility exercise for the thoracic spine was a high-intensity exercise against gravity. Thus, better results were obtained in the thoracic spine owing to the difference in intensity despite performing both exercises at the same time. Although the CVA measured from profile photographs also indicated improved results in both groups, in the standing position, there was a significant difference between groups through flow of time and there was an interaction. A previous study reported that thoracic spine mobilization with continuous passive stimulus increased joint mobility and helped in improving the somatosensory system [59]. Because of changes in these qualities and in the quantities of proprioception information, it was indicated that the improvement of spine alignment lead to the difference in CVA. However, the reason why there was no interaction in the sitting position was because the curve of the thoracic and lumbar spines consisted of slight flexion in a comfortable sitting position. Depending on the posture, the difference of spine alignment might be affected in the cervical spine.

The difference of the MDC and MCID of NPRS in both groups is noteworthy. In the present study, the average change score exceeded both the MDC and MCID values in the thoracic group. Although the results indicated that there was a significant within-group difference in the thoracic group, no significant within-group difference can be concluded in the cervical group. The difference between the groups in NPRS was 1.4 points, which exceeded the MCID, indicating the clinically significant effect of the thoracic spine mobilization and mobility exercise. It was, however, considered that the 95% CI (0.6, 2.3) of the difference included lower values than the MCID. Therefore, although the difference in improvement between groups was statistically significant, the clinical importance was uncertain when the interpretation was performed on the basis of the 95% CI. For the NDI, although the average change score was 12.2% in the thoracic group only, which exceeded the MDC, the average difference in change scores between the two groups was 7%, which was lower than the MCID of NDI [51]. Therefore, although the difference in improvement between groups for the NDI was again statistically significant, the gap could be of little importance clinically when the interpretation was performed on the basis of MCID. There was no interaction between the two groups in the pain sensitivity test of the upper trapezius muscle. The reason why the upper trapezius muscle was targeted was to identify the effect of position improvement after the treatment, because the tone of the upper trapezius muscle was increased and became tighter due to upper cross syndrome [60]. This study indicated a significant effect in both groups at 4 weeks after the treatment. The muscle tone of the upper trapezius was decreased by the change of posture through joint mobilization, and cervical instability was improved through the therapeutic exercise. However, there was no interaction between the two groups, and the MCID of PPT was not exceeded because the intervention for the cervical and thoracic spine in this study had no direct effect on the sensitivity of the upper trapezius muscle [49]. Although the mechanism of the effect of the manual therapy on neck pain was not clear, the sensitivity of the mechanical receptor might be changed through the application of continuous passive stimulus to soft tissue [38, 61], and the mechanical stress of the pain generator might be reduced by improving the biomechanical relationship of the cervical spine and the thoracic spine [62, 63]. In case of GRC, 68.8% of the participants in the thoracic group and 50% of the participants in the cervical group showed higher than +4 points, and the difference between the groups was significant. The patients’ satisfaction was affected by various factors such as individual pain and the belief and there may be inconsistencies in the results among participants owing to their different environments [64]. However, the intervention for the thoracic group was shown to improve FHP better than the intervention for the cervical group based on the GRC in this study, which indicated that all participants were simultaneously improved in terms of the specific therapy technique.

The results of this study suggest that the correct posture of the cervical spine is important when using touchscreen smartphones; however, the correct alignment of the thoracic spine should be prioritized, and clinical interventions including the cervical spine and thoracic spine should be applied. The most important limitation of this study is the short-term follow-up of 6 weeks and the small sample size. Although the sample size was set by using the effect size of a previous study, it is difficult to generalize the study results to all patients with neck pain from FHP. Moreover, it is difficult to generalize the intervention results of this study to male patients with mechanical cervical pain because the sample comprised only 9 men (23 women). However, we considered that the study results can be generalized to the average population because recent studies have proved that women have a higher rate of neck pain than men [65]. Future studies with a long-term follow-up and evaluating the placebo effect, by investigating three groups including a control group, should be conducted.

## Conclusion

This study demonstrated that individuals with FHP who received the combination of upper thoracic spine mobilization and mobility exercise demonstrated better overall short-term outcomes in terms of the CVA (standing position), cervical extension, NPRS, NDI, and GRC than those who received upper cervical spine mobilization and stabilization exercise. Future studies should examine the effectiveness of different types and dosages of manual therapy, and perform long-term follow-up data collection.

## Abbreviations

ANOVA: Analysis of variance
CROM: Cervical range of motion
CVA: Craniovertebral angle
FHP: Forward head posture
GRC: Global rating of change
NDI: Neck disability index
NPRS: Numeric pain rating scale
PPT: Pressure pain threshold
